# Vibrational Spectroscopy of Peritoneal Dialysis Effluent for Rapid Assessment of Patient Characteristics

**DOI:** 10.3390/biom10060965

**Published:** 2020-06-26

**Authors:** Tom Grunert, Rebecca Herzog, Florian M. Wiesenhofer, Andreas Vychytil, Monika Ehling-Schulz, Klaus Kratochwill

**Affiliations:** 1 Functional Microbiology, Institute of Microbiology, Department of Pathobiology, University of Veterinary Medicine, Veterinaerplatz 1, 1210 Vienna, Austria; monika.ehling-schulz@vetmeduni.ac.at; 2 Christian Doppler Laboratory for Molecular Stress Research in Peritoneal Dialysis, Department of Pediatrics and Adolescent Medicine, Medical University of Vienna, 1090 Vienna, Austria; rebecca.herzog@meduniwien.ac.at (R.H.); florian.wiesenhofer@meduniwien.ac.at (F.M.W.); 3 Division of Pediatric Nephrology and Gastroenterology, Department of Pediatrics and Adolescent Medicine, Comprehensive Center for Pediatrics, Medical University of Vienna, 1090 Vienna, Austria; 4 Division of Nephrology and Dialysis, Department of Medicine III, Medical University of Vienna, 1090 Vienna, Austria; andreas.vychytil@meduniwien.ac.at

**Keywords:** peritoneal dialysis, peritoneum, vibrational spectroscopy, FTIR, molecular signatures, peritonitis, metabolites, machine learning, photonic-based diagnostics

## Abstract

Peritoneal dialysis (PD) offers specific advantages over hemodialysis, enabling increased autonomy of patients with end-stage renal disease, but PD-related complications need to be detected in a timely manner. Fourier transform infrared (FTIR) spectroscopy could provide rapid and essential insights into the patients’ risk profiles via molecular fingerprinting of PD effluent, an abundant waste material that is rich in biological information. In this study, we measured FTIR spectroscopic profiles in PD effluent from patients taking part in a randomized controlled trial of alanyl-glutamine addition to the PD-fluid. Principal component analysis of FTIR spectra enabled us to differentiate between effluent samples from patients immediately after completion of instillation of the PD-fluid into the patients’ cavity and 4 h later as well as between patients receiving PD-fluid supplemented with 8 mM alanyl-glutamine compared with control. Moreover, feasibility of FTIR spectroscopy coupled to supervised classification algorithms to predict patient-, PD-, as well as immune-associated parameters were investigated. PD modality (manual continuous ambulatory PD (CAPD) vs. cycler-assisted automated PD (APD)), residual urine output, ultrafiltration, transport parameters, and cytokine concentrations showed high predictive potential. This study provides proof-of-principle that molecular signatures determined by FTIR spectroscopy of PD effluent, combined with machine learning, are suitable for cost-effective, high-throughput diagnostic purposes in PD.

## 1. Introduction

Patients with end-stage renal disease depend on life-saving dialysis for removal of substances, which are usually subject to urinary excretion, such as salts and water from the uremic body. Peritoneal dialysis (PD) offers specific advantages compared with hemodialysis, such as, for instance, more freedom from hospital-based dialysis centers, better preservation of residual renal function, and an advantage in survival at the beginning of renal replacement therapy [1,2]. Unfortunately, these benefits are counterbalanced by its limitations, including the risk of peritonitis, inflammation, and membrane failure as the main physiological culprits of technique failure [3]. In particular, the occurrence of bacterial infections requires rapid and accurate decisions based on profound diagnostics.

Management of PD patients currently mainly relies on systemic parameters—which might be diagnostically inconclusive, too late, and/or too unspecific—and the peritoneal equilibration test (PET), a functional test of peritoneal transport. However, regular performance of PETs is laborious; time-consuming; and, if performed more often than the recommended 12-month interval to closely monitor the patients, also a cost-intensive way of guiding the therapy regime of PD patients. Long-term surveillance of patients on home therapy, having only intermittent routine checks at the tertiary care center, is also a challenge in PD patients, sometimes leading to delayed discovery of complications.

PD effluent contains significant information on peritoneal biology and the state of the membrane in the form of dissolved molecules. However, PD effluent samples from high-risk and low-risk patients are hardly distinguishable based on routine lab measurements. These limitations of current routine diagnostic tests resulted in an increased interest in novel technical approaches to provide clinicians with better tools to manage the dialysis regime or to detect acute infectious events of hospitalized PD patients [4,5]. The generation of omics profiles might be an attractive approach to characterize patients in different stages of disease and therapy. The advent of omics methods has enabled a deeper characterization of PD effluents at the level of proteins, metabolites, and cells using a variety of analytical methods, such as microarray and sequencing techniques, liquid chromatography-electrospray ionization (LC-ESI)-, and matrix-assisted laser desorption/ionization (MALDI)- mass spectrometry (MS) (including 2D gel-based) techniques [4,6,7,8,9,10,11]. However, hitherto none of these molecules has yet been accepted as a predictive biomarker [4]. Although omics methods allow broader biological insight, they are substantially more expensive and time-consuming than routinely used clinically applicable diagnostic tests. So far, no rapid method for analyzing a PD patient’s biofluid, which would enable to assign clinical phenotypes in order to provide clinicians with better tools to monitor/manage dialysis regime and/or to predict the PD outcome, has been described and is thus urgently needed.

Over the last decade, photonic-based analytics including vibrational spectroscopy developed from a niche activity into a key enabling technology (KET), becoming one of the most important industries for the future [12]. Vibrational spectroscopy techniques, such as Fourier-transform infrared (FTIR) spectroscopy are rapid, non-invasive, and cost-effective analytical tools with high-throughput capacities. These techniques have already successfully been employed to discriminate between normal and pathological populations within different tissue and/or cell types as well as biofluids, such as blood-plasma/serum, urine, saliva, and synovial fluid [13,14]. For instance, the potential of FTIR spectroscopy has been shown to be suitable for diagnosing and monitoring the progression of ovarian cancer, screening for colorectal cancer, and Alzheimer’s disease, based on blood-plasma/serum samples and/or peripheral blood mononuclear cells [15,16,17]. In the context of hemodialysis, urea, triglycerides, glucose, phosphate, cholesterol, albumin, total protein, and the extent of lipid peroxidation were determined in plasma and in dialysate by FTIR spectroscopy [18,19,20].

PD effluent, a readily accessible biofluid, can be considered ideal for routine clinical implementation of FTIR spectroscopy. During measurement, a spectral signature based on all biochemical constituents within a sample is generated, also referred to as molecular spectral fingerprint [21]. In biofluids, the most important spectral regions can be assigned to functional groups of the major biomolecules including polysaccharides, proteins, and fatty acids (for detail, see Table 1) [22,23]. Using a diverse array of feature extraction methods, the sample-specific spectroscopic signature can be subsequently determined and used to classify samples by means of machine learning [24]. Compared with conventional tests in clinical chemistry, machine learning-assisted spectroscopy has the advantage that it provides a “holistic view” of a sample via the simultaneous investigation of a range of different molecules instead of identifying and quantifying specific compounds. Because of its high-throughput capacity at low running costs (reagent-free, label-free, reusable sample holders), vibrational spectroscopy is perfectly suited for large-scale studies as well as for implementation in routine analytics.

So far, the use of FTIR spectroscopy in the analytical context with PD effluent samples from patients has not been reported. In this pilot study, we investigated the potential of machine learning-assisted FTIR spectroscopy as a tool to assess the individual patient’s status and to evaluate possible clinical interventions. Further, we examined the correlation of FTIR spectroscopic molecular signatures to specific compositional properties obtained by means of targeted metabolomics by mass spectrometric analysis.

## 2. Materials and Methods

### 2.1. Patients and Ethics

PD effluent samples were obtained during a prospective randomized, open-label, two-period, cross-over phase I/II study conducted at the Medical University of Vienna (Austria) at the Department of Nephrology. The study and study protocol were approved by the local ethics committee of the Medical University of Vienna (EK 867/2010, EK 2035/2015) and registered at www.clinicaltrials.gov (NCT01353638). The study was performed in accord with the Declaration of Helsinki. All patients provided written informed consent prior to trial participation. The study design, eligibility criteria, randomization, clinical methods, patient characteristics, and adverse events have been previously described [10]. In brief, 20 stable PD patients (13 male/7 female, median age 58 (47–68) years) with a mean PD vintage of 2.4 years were treated per protocol. Patients were judged as clinically stable and had no severe concomitant disease (five patients had a history of peritonitis more than 3 months prior to sample collection).

Effluent samples were collected in standard collection tubes without any anticoagulants (Vacuette, Bio-Greiner-One, Kremsmünster, Austria) immediately after completion of instillation of the dialysis fluid into the patients’ cavity (= time point 0) and 4 h later (= time point 4 h) after each of two standard peritoneal equilibration tests (PETs), one using commercially available PD fluid (Dianeal, 3.86% glucose, Baxter, Deerfield, IL, USA) and a second with the same PD fluid supplemented with 8 mM alanyl-glutamine (Ala-Gln) (N(2)-alanyl-L-glutamine 200 mg/mL, Fresenius Kabi, Bad Homburg, Germany), performed in randomized order. The two PETs were separated by a wash-out period of 28 to 35 days.

### 2.2. Sample Collection and Preparation

PD effluent was centrifuged (250× *g*, 10 min) immediately following collection, and cell-free supernatant samples were aliquoted and stored at −80 °C until further analysis. Biochemical measurements and transfer kinetics between peritoneal and systemic circulation were analyzed in serum (at 2 h of PET) and dialysate specimens by standard methods in the clinical laboratory of the Vienna General Hospital. PD effluent concentrations of creatinine were determined by a kinetic measurement of Jaffé reaction and corrected for high glucose levels by determination of a correction factor from measurements of unused PD fluid with the same method. PD effluent interleukin 6 (IL-6) and interleukin 8 (IL-8) concentrations were measured with the Immulite system (Siemens, Vienna, Austria) or by Bio-Plex bead array (Bio-Rad, Hercules, CA, USA).

### 2.3. FTIR Spectroscopy

#### 2.3.1. Sample Preparation and FTIR Spectroscopy

PD effluent samples were arranged in randomized order and were measured and analyzed blinded. PD effluent samples were thawed and 30 µL of undiluted or 1:2 dilutions in 0.9% NaCl was subsequently spotted on a zinc selenide (ZnSe) optical plate and dried at 40 °C for 30 min. Infrared spectral acquisition was performed in transmission mode using an microplate adapter (HTS-XT) coupled to a Tensor 27 FTIR spectrometer (Bruker Optics GmbH, Ettlingen, Germany) using the following parameters: 4000 to 500 cm^−1^ spectral range, 6 cm^−1^ spectral resolution, zero-filling factor of 4, Blackmann-Harris three-term apodization, and 32 interferograms were averaged with background subtraction for each spectrum [25]. In total, 79 spectra (0 h effluents, *n* = 39; 4 h effluents, *n* = 40) were recorded derived from PD effluent samples of 20 patients.

#### 2.3.2. Spectral Preprocessing and Analysis

Spectral preprocessing and subtractive spectral analysis were performed using the software OPUS 7.2 (Bruker Optics GmbH). Spectral preprocessing was performed to increase spectral resolution, to minimize baseline shifts, and to adjust biomass variations among different sample preparations. Raw absorbance spectra were preprocessed for the whole spectral range (4000–500 cm^−1^) either (1) by vector normalization followed by baseline correction or (2) by second derivatives of the original spectra with a nine-smoothing point Savitzky–Golay filter followed by vector normalization; the latter method was used for the calculation of subtractive spectra and unsupervised and supervised machine learning [26,27].

#### 2.3.3. Unsupervised and Supervised Machine Learning

All multivariate analyses were performed using the software Unscrambler X (CAMO Software, Oslo, Norway). Unsupervised machine learning was performed on preprocessed data employing principal component analysis (PCA). The method refers to “unlabeled” data, where the classification is not included in the observations and allows a picture of the inherent data structure by reducing the data complexity. PCA computation was based on the singular value decomposition (SVD) algorithm and performed at the spectral range of either proteins (1800–1500 cm^−1^) or polysaccharides (1200–800 cm^−1^). The score plot was used for explorative data analysis and shows possible discriminations between classes, whereas the loading plot provides information about relevant spectral regions [27].

Supervised machine learning was performed on preprocessed data employing principal component analysis-linear discriminant analysis (PCA-LDA) and principal component analysis-Mahalanobis discriminant analysis (PCA-MDA). The linear method of discriminant analysis (LDA) is applied if the difference between the two classes is expressed by a linear function, whereas the Mahalanobis method (MDA) uses ellipses to define the distances. For PCA-LDA and PCA-MDA, the following parameters were used: seven components for PCA projection (capturing more than 98% of the cumulative explained variance), assume equal prior probabilities, and all variables have equal weights. Cross validation was performed for the combined data set (*n* = 40) including both treatments without and with Ala-Gln after 4 h dwell time to obtain sufficient amount of data per group to run PCA-LDA and PCA-MDA analysis. Data were divided into ten cross-validation segments.

All available numeric datasets were divided into two balanced groups and transformed in category variables to achieve a simple binary classification problem. The classification rate (%) was used as a classification performance measure, which is defined as the average between the individual accuracy (%) of each of the two classes, which applies for imbalanced datasets instead of counting the overall correct guesses [16].

### 2.4. Targeted Metabolomics

Analysis of metabolites was performed as described before [11]. In brief, 188 metabolites were measured using the AbsoluteIDQ p180 kit (Biocrates Life Science AG, Innsbruck, Austria) by LC/FIA-MS. Raw-data processing and validation were performed by the MetIDQ software (Biocrates). All further data analysis was performed in R (www.R-project.org). The data were filtered for metabolites with at least 75% valid data points (above the respective LOD) in the 4 h effluents, resulting in 92 metabolites included in this study. One sample with missing values was excluded.

Metabolomics data obtained from the 4 h effluents of the control group were correlated with signals from each wavenumber of the corresponding FTIR data within the range of 3000–2800 cm^−1^ and 1800–800 cm^−1^, and Pearson’s correlation coefficients were calculated. The obtained correlation profiles of metabolites were hierarchically clustered using complete linkage and Euclidian distances. Amino acid and biogenic amine concentrations from both time points and treatments were z-scaled prior to PCA.

## 3. Results and Discussion

### 3.1. Spectral Impact on PD Fluid during Dialysis and Owing to Ala-Gln Treatment

**Figure 1:**
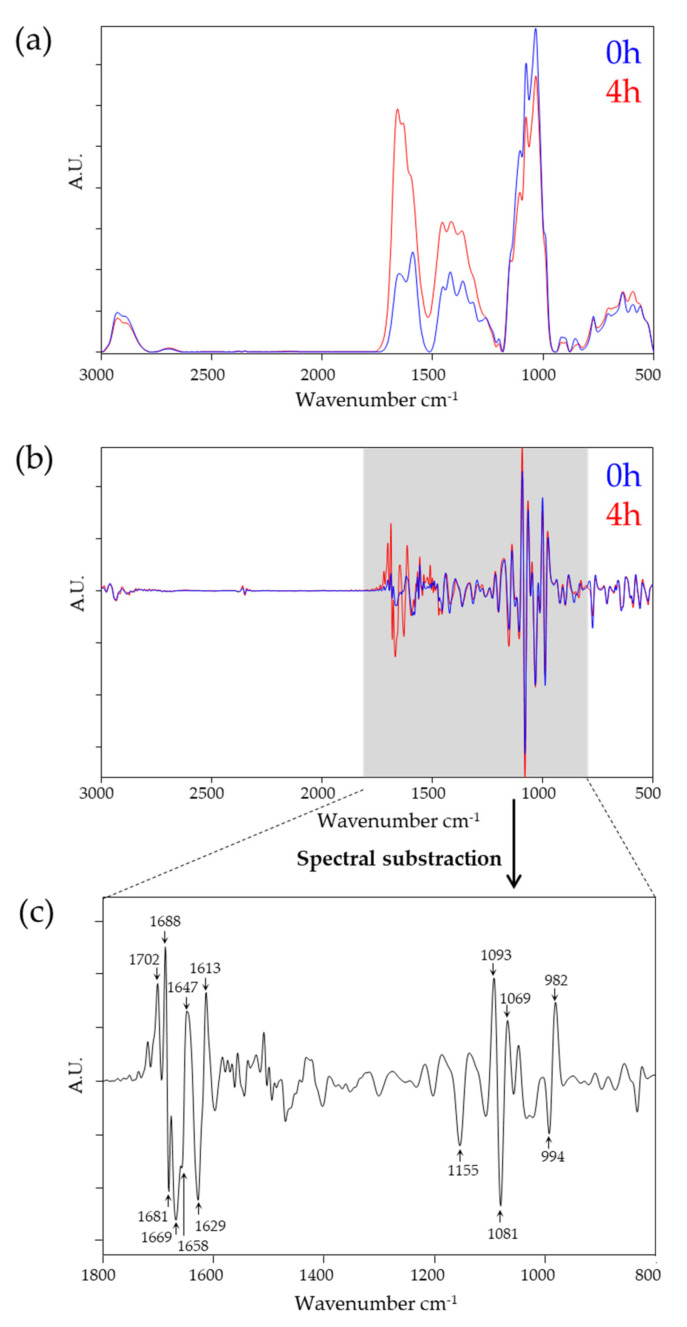
Spectral comparison of 0 h and 4 h peritoneal dialysis (PD) effluent samples. (**a**) Average spectra of vector-normalized, baseline corrected spectra (0 h, *n* = 39; 4 h, *n* = 40). (**b**) Average spectra of second derivative, baseline corrected spectra (0 h, *n* = 39; 4 h, *n* = 40). (**c**) Subtraction spectra generated of second derivative, baseline corrected spectra at the relevant spectral range of 1800–800 cm^−1^. Spectra from 4 h were subtracted from 0 h. Wavenumbers responsible for discrimination are indicated. A.U., arbitrary units.

First, we evaluated the differences between 0 h and 4 h PD effluent sampling time points. Effluent samples obtained immediately after completion of instillation of the dialysis fluid into the patients’ cavity (= time point 0 h) and at the end of the 4 h equilibration time (= time point 4 h) are expected to represent different chemical composition resulting from osmotic transport processes. Figure 1a shows average, vector normalized, and baseline corrected absorbance spectra of 0 h and 4 h PD samples covering the spectral range of 3000–500 cm^−1^. The 4 h samples showed an increase in peak area particularly in the protein (1800–1500 cm^−1^) spectral region, whereas the polysaccharide region (1200–900 cm^−1^) remained nearly unchanged compared with time point 0 h samples. Thus, as expected from the clinical routine laboratory data, owing to the transition of protein compounds as well as uremic toxins from the patients’ peritoneum into the PD effluent, the increased protein/peptide content in the PD effluent after 4 h dwell time could be observed in the spectra.

Second-order derivative and vector-normalized spectra were used to qualitatively examine the spectra in more detail. The average spectrum of 0 h samples was subtracted from the spectrum of 4 h samples (Figure 1b,c, respectively). Significant differences between the two sampling points were obtained within the (i) protein region (1720–1600 cm^−1^) and (ii) polysaccharides region (1200–900 cm^−1^). In particular, the observed spectral changes at 1702, 1688, 1681, 1669, 1647, 1629, and 1613 cm^−1^ can be assigned to changes in the conformation sensitive amide I band (1690–1620 cm^−1^). The amide I band originates from symmetric stretching vibrations of the carbonyl (C=O) functional group and indicates changes in anti-parallel sheets and β-turn (1688, 1681, 1669 cm^−1^), α-helical (1657 cm^−1^), unordered (1647 cm^−1^), and β-plated sheet structures (1629 cm^−1^) [23,28]. The most prominent differences in the polysaccharide region were located at 1155, 1093, 1081, 1069, 994, and 982 cm^−1^. These spectral ranges are dominated by vibrations of various oligo- and polysaccharides including the glycosidic linkages type and around 1081 cm^−1^ by ‘phosphorus-containing biomolecules’, detecting vibrations of phosphodiester functional groups of DNA/RNA and/or phospholipids or phosphorus-containing carbohydrates [23,28].

Although it has recently become technically feasible to analyze the dialysate composition on the protein level, the relevance of individual proteins in the effluent is still far from understood [8]. Even less is known on the polysaccharide level. Our method, however, enables correlation of these signals to clinical outcome as well as measurements from individual biomarker molecules.

**Figure 2:**
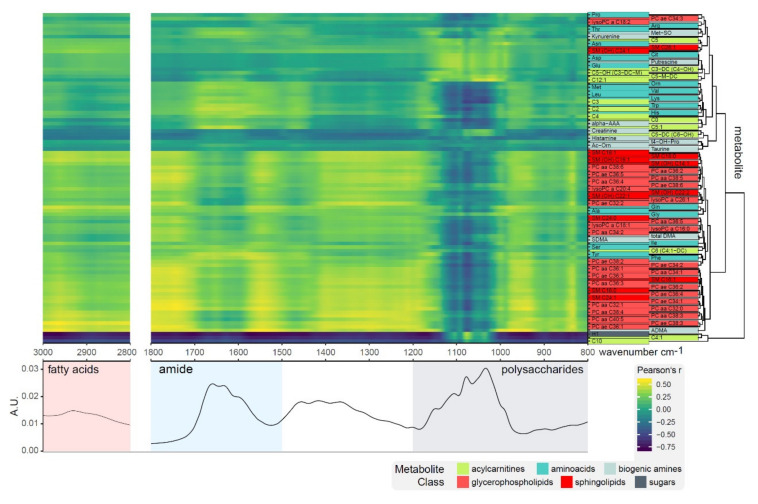
Correlation profiles of metabolite concentrations with fatty acid, amide, and polysaccharide regions of the FTIR spectra for 4 h control samples. Clustering of metabolites revealed three distinct groups of small molecules; a cluster containing glucose correlating well with the polysaccharide band (1200–1000 cm^−1^); a second cluster primarily made up of lipids correlating well with the lipid region (3000–2800 cm^−1^); and a third cluster of amino acids, biogenic amines, and acylcarnitines.

Correlation profiles of 92 metabolites with the spectral regions of fatty acids, amides and polysaccharides revealed three noticeable clusters: (1) a hydrophilic cluster with very diverse sub-clusters containing mostly amino acids, biogenic amines, and acylcarnitines; (2) a lipophilic cluster containing mainly glycerophospholipids and sphingolipids, showing good correlation with the fatty acid region (3000–2800 cm^−1^); and (3) hexoses and two acylcarnitines correlating well with the polysaccharide region (1200–1000 cm^−1^) (Figure 2). The high chemical diversity of metabolites in cluster 1 is apparent from the dissimilarity of correlation profiles. Proline (Pro) to sphingolipid SM(OH)-C24:1 show the most diverse profiles, citrulline (Cit) to dodecenoylcarnitine (C12:1) correlate with various parts of the polysaccharide region, ornithine (Orn) to tiglylcarnitine (C5:1) correlate well within the amide region, and creatinine to taurine do not seem to correlate with the spectral information.

**Figure 3:**
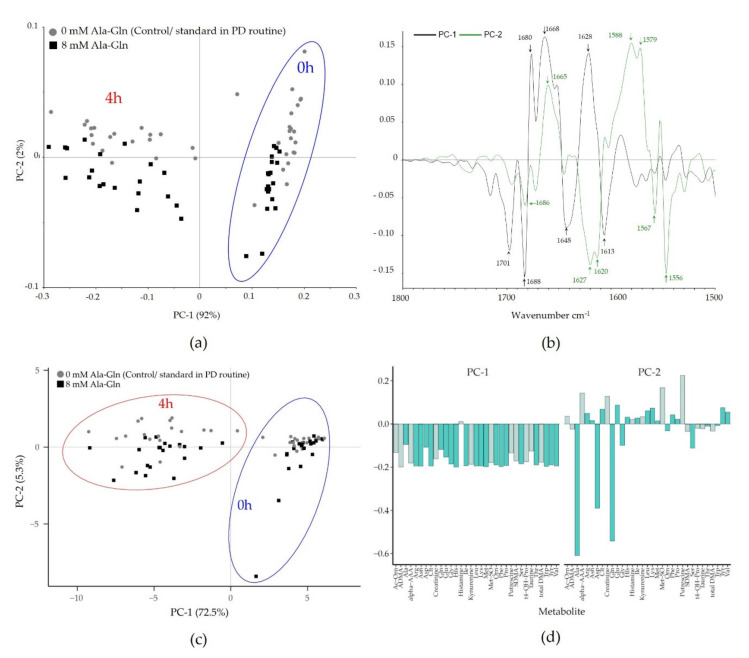
Unsupervised principal component analysis (PCA) for discrimination between 0 h and 4 h as well as Ala-Gln treated and untreated patients in Fourier-transform infrared (FTIR) spectroscopic and targeted metabolomics data. (**a**) PCA score plot from FTIR data based on the protein spectral region (1800–1500 cm^−1^). PC1 was plotted against PC2. PC1 shows a distinct clustering between spectra from 0 h and 4 h derived samples, whereas PC2 discriminates the spectra of Ala-Gln treated and non-treated patients. The equivalent score plot for the polysaccharide region is provided in the Figure S1. (**b**) PCA loading plot identifies the Amide I region (1690–1620 cm^−1^) to be mainly responsible between the spectra of the 0 h and 4 h sampling points. The Amide II region (1570–1515 cm^−1^) also accounts for the separation between Ala-Gln untreated vs. treated PD samples. (**c**) In the targeted metabolomics data, PCA of 92 metabolites were included. The first PC represents 72.5% of the variation, followed by 5.3% by PC2. The biplot shows separation by dwell time, but not by treatment. (**d**) PCA loading plot indicates a uniform influence of most features on PC1, whereas the loadings of PC2 show a separation mainly by Ala, Gln, and Asp (dark green, canonical AA; gray, biogenic amines).

PCA was employed to investigate the discriminatory features of the two sampling time points (0 h and 4 h) and the Ala-Gln treatment of both FTIR and targeted metabolomics data. For the FTIR data, the score plot for PC1 revealed a clear clustering of spectral data according to the sampling time, whereas PC2 separated spectra of PD samples derived from patients with or without Ala-Gln treatment using the spectral range of the protein region (1800–1500 cm^−1^) (Figure 3a). The variance explained by these two principal components, however, is in a completely different range (PC1 92%, PC2 2%), showing that the sample composition changes completely within 4 h dwell time, but the differential effect of Ala-Gln supplementation on the overall composition is subtle. Figure 3b shows the loading plot generated to identify the spectral bands particularly contributing to these differences. In line with the result from the spectral comparison, the Amide I region (1690–1620 cm^−1^) mainly accounts for differences between spectra of the 0 h and 4 h sampling time points. Interestingly, the separation between the spectra derived from 0 mM versus 8 mM Ala-Gln supplemented PD fluid samples relies additionally on the Amide II region (1570–1515 cm^−1^). Performing PCA based on the spectral region of polysaccharides (1200–800 cm^−1^) showed a clear clustering between spectra of 0 and 4 h sampling points, Ala-Gln-supplemented and not-supplemented samples could not be separated (Figure S1).

For the targeted metabolomics data, the PCA of the metabolites shows a clear separation by dwell time, but less so by Ala-Gln treatment (Figure 3c,d). The loadings for PC1 indicate a uniform influence of most features on the PCA for the targeted metabolomics data. Interestingly, the loadings of the second PC show a separation of loadings by Ala, Gln, and Asp, which in turn correlates very well with the obtained separation of Ala-Gln treated/untreated-samples in PC2 by FTIR spectroscopy (Figure 3a). Although the overall pattern is in accordance between the two analytical techniques, FTIR data more clearly separate controls and Ala-Gln-treated samples. In contrast to a direct and unambiguous assessment of analyte concentrations, as obtained from targeted metabolomics, FTIR spectra show a more global change to analyte concentration changes in many wavenumbers, and might thus better detect even subtle changes in the nature of these samples. Thus, this study also shows that the combination of FTIR spectroscopic molecular fingerprinting and in-depth metabolomics analysis is of utmost value, helping to better interpret the molecular signatures of complex biological samples obtained by FTIR spectroscopy [29,30], providing a route for future research in integrated metabolic modelling.

### 3.2. Supervised Machine Learning of Patient-, PD-, and Infection and Inflammation-Related Parameters

As we could successfully discriminate between sampling time points and treatment with and without Ala-Gln using FTIR, we headed towards testing predictive models capable to distinguish patient-, PD-, and infection and inflammation-related parameters. The aim of this analysis was not to provide ready-to-use clinical stratification algorithms, but to roughly investigate if patient sub-groups can in principle be discriminated on the level of their molecular spectral signatures. Classification of spectroscopic data was performed using either the highly discriminatory protein (1800–1500 cm^−1^) or polysaccharide (1200–800 cm^−1^) spectral region, applying the following supervised learning based classification algorithms: principal component analysis-linear discriminant analysis (PCA-LDA) and principal component analysis-Mahalanobis discriminant analysis (PCA-MDA). Owing to the limited size of the sample cohort, two different data sets were used: either (1) PD effluent samples without Ala-Gln after 4 h dwell time (*n* = 20) to reflect conditions during daily PD routine, or (2) samples from both treatments without and with Ala-Gln after 4 h dwell time were combined (*n* = 40) to obtain sufficient amount of data per group to run PCA-LDA and PCA-MDA analysis including cross-validation. Thus, the latter analysis (*n* = 40) may be influenced by additional factors owing to Ala-Gln treatment.

All results were plotted as a confusion matrix (Tables S1–S3) and the classification rates are summarized in Table 2.

Classification results based on FTIR-spectroscopic data related to the patient characteristic, age, and sex are shown in Table 2A. High classification rates (≥95%) were obtained for patient age (higher 60/below 60) using PCA-LDA/PCA-MDA in either the protein or polysaccharide spectral region. Discrimination between PD samples derived from female and male patients revealed no favorite spectral range or classifier and had a maximum of 78/66.7% total accuracy (*n* = 20/40, PCA-LDA/PCA-MDA at the protein region). Thus, FTIR spectroscopic data modelling provides evidence for the existence of an age-related molecular signature of PD effluent samples capable to discriminate between patients younger or older than 60 years.

Next, supervised classification was employed to correlate the PD-related parameters, such as continuous ambulatory PD (CAPD)/automated PD (APD)-cycler modality, use of glucose/icodextrin solutions, time on PD, residual urine output (UrinVolOut), ultrafiltration (UF), residual clearance (ResidualCl), and dialysate-to-plasma creatinine ratio (D/P crea). The highest classification rates were achieved for therapy modality (95.0%), D/P crea (100.0%), UF (100.0%), and glucose/icodextrin (81.3%) in the polysaccharide region, whereas the protein region separated best for UrinVolOut (100.0%), ResidualCl (89.0%), and time on PD (68.3%) sample parameters (Table 2B). The assignment of data related to the PD vintage, such as the therapy modality (95.0%), may be relevant for patient stratification and understanding of clinical phenotypes. Assignment for (patho)physiological parameters may be of diagnostic value. The highest classification rates were achieved for assignment of D/P creatinine ratio (100.0%) and UF (100.0%) in the polysaccharide region, whereas the protein region separated best for residual urine output (100.0%) and residual clearance (89.0%). Parameters such as ultrafiltration and dialysate-to-plasma creatinine ratio are currently determined by the time extensive PET. Assessment of residual urine output and residual clearance requires further collection of urine. It is, of course, desirable to obtain similar information faster, for example, by spectroscopic analysis of PD effluent.

The same algorithms were applied to test correlation between PD spectral features, parameters of inflammation (IL-6 and IL-8 in the cell-free PD effluent supernatant), and stress responses (cellular abundance of heat shock protein 72 (HSP-72)), as well as a history of previous peritonitis, all of which have been assessed in this material in the original study [10]. As shown in Table 2C, prediction of IL-6 achieved an 85.7% classification rate using the protein region. Discrimination between samples derived from patients with or without previous peritonitis achieved 66.7% for the polysaccharide spectral region. The concentration of biomarkers can be obtained using traditional methods and some individual biomarkers have recently been shown to correlate with peritoneal membrane status (e.g., IL-6 effluent concentrations and peritoneal small solute transport rate (PSTR) [31]). The spectrum-based assignment, however, may provide an easier way of obtaining holistic biomarker information.

There is a significant diagnostic potential of the combination of machine-learning assisted FTIR spectroscopy and analysis of biofluids for predicting the efficacy of clinical interventions. This study provides a promising starting point for using FTIR spectroscopy in PD diagnostics. Owing to limitations of this study with respect to sample size and potential bias related to the nature of the clinical trial cohort, further validation of the method is indicated before it can be implemented in routine diagnostics. Metabolites from food, drugs, or other exogenous factors may end up in the peritoneum and contribute to the PD effluent FTIR signature. It could thus be relevant for future studies to collect data on nutritional status and current medication in order to try to integrate these data into the models. Our proof-of-principle study provides the basis for further studies with larger patient cohorts and more samples to explore possible new features of diagnostic value and to further improve and validate the performance of our photonic-based PD effluent diagnostic method.

## 4. Conclusions

This pilot study provides proof-of-principle that PD effluent samples-based FTIR spectroscopy combined with machine learning might be suitable in daily PD routine diagnostics for rapid, cost-effective point-of-care clinical monitoring of the patient status and risk profile. The advantage of combining FTIR spectroscopy and PD effluents is that complicated sample preparation steps are eliminated/not required. The correlation of FTIR spectroscopic data to targeted metabolomics data confirmed the anticipated molecular features of PD effluent as the responsible components for specific spectral regions. Once sufficient training data are available, the application of FTIR spectroscopy in clinical routine might offer a promising, cost-effective alternative to obtain information that otherwise requires laborious and expensive functional tests. Longitudinal data collection from the same patient might help to reduce the influence of inter-individual variation, as data points from the same patient are compared over time. The use of improved machine learning and/or pattern recognition algorithms trained with a larger PD sample data set will further improve the performance of these photonic-based PD effluent diagnostics.

## 5. Patents

Patent application resulting from the work reported in this manuscript: Grunert T., Kratochwill K.; spectroscopy-based analysis in peritoneal dialysis. Filed at EP (EP19193619.4): 26 August 2019.

## Figures and Tables

**Table 1:** Assignment of important Fourier-transform infrared (FTIR) spectral regions of biofluids.

Frequency (cm^−1^)	Spectral Region	Assignment
3000–2800	Fatty acids	dominated by fatty acid chains (e.g., phospholipids)
1800–1500	Proteins	dominated by conformation-sensitive amide I and amide II bands of proteins and peptides
1500–1200	Proteins and fatty acids (’mixed region’)	a region with complex absorption profiles from proteins as well as fatty acids
1230/1085	Phosphorus-containing biomolecules	detects vibrations of, for example, phospholipids or other phosphorus-containing biomolecules
1200–800	Polysaccharides	dominated by vibrations of various oligo- and poly-saccharides and their specific type of glycosidic linkages

**Table 2:** Classification rates (%). The gray scale is according to the classification rates to instantly overview the top-performing combinations of all variable data parameters (classification rates: ≥ 70%, light gray; ≥ 80%, gray and ≥ 90%, dark gray).

	Protein Region		Polysaccharide Region	
	(1800–1500 cm^−1^)		(1200–800 cm^−1^)	
**(A) Patient-related data**	**LDA**	**MDA**	**LDA**	**MDA**
**Age (<60/≥60 years)**				
control, 4 h (*n* = 20)	80.0	100.0	95.0	100.0
all, 4 h (*n* = 40)	70.0	67.5	72.5	60.0
**Sex (female/male)**				
control, 4 h (*n* = 20)	78.0	64.3	78.0	71.4
all, 4 h (*n* = 40)	45.8	66.7	45.8	49.0
**(B) PD-related data**	**LDA**	**MDA**	**LDA**	**MDA**
**Modality (CAPD/APD-Cycler)**				
control, 4 h (*n* = 20)	80.0	90.0	95.0	95.0
all, 4 h (*n* = 40)	87.5	77.5	77.5	82.5
**Glucose/Icodextrin**				
control, 4 h (*n* = 20)	n.d.	n.d.	n.d.	n.d.
all, 4 h (*n* = 40)	64.1	50.0	81.3	50.0
**Time on PD (<1/≥1 year)**				
control, 4 h (*n* = 20)	n.d.	n.d.	n.d.	n.d.
all, 4 h (*n* = 40)	68.3	35.0	66.7	46.7
**UrinVolOut (</≥1000 mL)**				
control, 4 h (*n* = 20)	93.8	100.0	75.0	93.8
all, 4 h (*n* = 40)	69.8	70.8	62.5	66.7
**UF (</≥550 mL)**				
control, 4 h (*n* = 20)	75.0	95.0	90.0	100.0
all, 4 h (*n* = 40)	63.6	62.4	65.2	56.8
**ResidualCl (</≥3 mL/min/1.73 m^2^)**				
control, 4 h (*n* = 20)	89.0	85.7	78.0	57.1
all, 4 h (*n* = 40)	70.7	71.3	50.7	65.3
**DP Crea (</≥ 0.8 (-))**				
control, 4 h (*n* = 20)	85.4	95.5	94.4	100.0
all, 4 h (*n* = 40)	76.7	65.3	71.3	78.0
**(C) Immunesystem-related data**	**LDA**	**MDA**	**LDA**	**MDA**
**IL-8 (</≥3 pg/mL)**				
control, 4 h (*n* = 20)	66.2	83.3	66.2	83.3
all, 4 h (*n* = 40)	45.3	50.3	54.7	44.4
**IL-6 (</≥200 pg/mL)**				
control, 4 h (*n* = 20)	85.7	50.0	78.0	50.0
all, 4 h (*n* = 40)	70.8	57.7	67.9	57.7
**Hsp-72 (</≥0.5 AU)**				
control, 4 h (*n* = 20)	100.0	100.0	79.8	100.0
all, 4 h (*n* = 40)	63.4	44.5	50.3	59.8
**Previous peritonitis (yes/no)**				
control, 4 h (*n* = 20)	n.d.	n.d.	n.d.	n.d.
all, 4 h (*n* = 40)	53.3	48.3	66.7	51.7

Control: standard fluid, all: control and treatment with standard fluid +Ala-Gln; CAPD: continuous ambulatory peritoneal dialysis; APD-cycler: automated PD; UrinVolOut: residual urine output; UF: ultrafiltration; ResidualCl: residual clearance; IL: interleukin; D/P Crea: dialysate-to-plasma creatinine ratio; n.d.: not-determinable; Hsp: heat shock protein; LDA, linear discriminant analysis; MDA, Mahalanobis discriminant analysis.

## Supplementary Materials

The following are available online at https://www.mdpi.com/2218-273X/10/6/965/s1, Figure S1: PCA score plot from FTIR data based on the polysaccharide spectral region (1200–800 cm^−1^); Tables S1–S3: Confusion matrix incl. classification rates of (S1) patient-related data (balanced), (S2) PD-related data (balanced), and (S3) immune system-related data (balanced).
