# Supplementary material for: Vibrational Spectroscopy of Peritoneal Dialysis Effluent for Rapid Assessment of Patient Characteristics
Source: Biomolecules. 2020 Jun 26;10(6):965. doi: 10.3390/biom10060965 (PMC7357123; doi:10.3390/biom10060965)
Supplement: Supplementary file 1 [file biomolecules-10-00965-s001.pdf]

biomolecules

MDPI

Supplementary Materials:

Vibrational Spectroscopy of Peritoneal Dialysis Effluent for Rapid Assessment of Patient Characteristics

Tom Grunert [1,*,†], Rebecca Herzog [2,3], Florian M. Wiesenhofer [2,3], Andreas Vychytil [4], Monika Ehling-Schulz [1], and Klaus Kratochwill [2,3,*,†]

[1] Functional Microbiology, Institute of Microbiology, Department of Pathobiology, University of Veterinary Medicine, Veterinaerplatz 1, 1210 Vienna, Austria
[2] Christian Doppler Laboratory for Molecular Stress Research in Peritoneal Dialysis, Department of Pediatrics and Adolescent Medicine, Medical University of Vienna, 1090 Vienna, Austria
[3] Division of Pediatric Nephrology and Gastroenterology, Department of Pediatrics and Adolescent Medicine, Comprehensive Center for Pediatrics, Medical University of Vienna, 1090 Vienna, Austria
[4] Division of Nephrology and Dialysis, Department of Medicine III, Medical University of Vienna, 1090 Vienna, Austria
* Correspondence: tom.grunert@vetmeduni.ac.at (T.G.); klaus.kratochwill@meduniwien.ac.at (K.K.)
† Contributed equally.

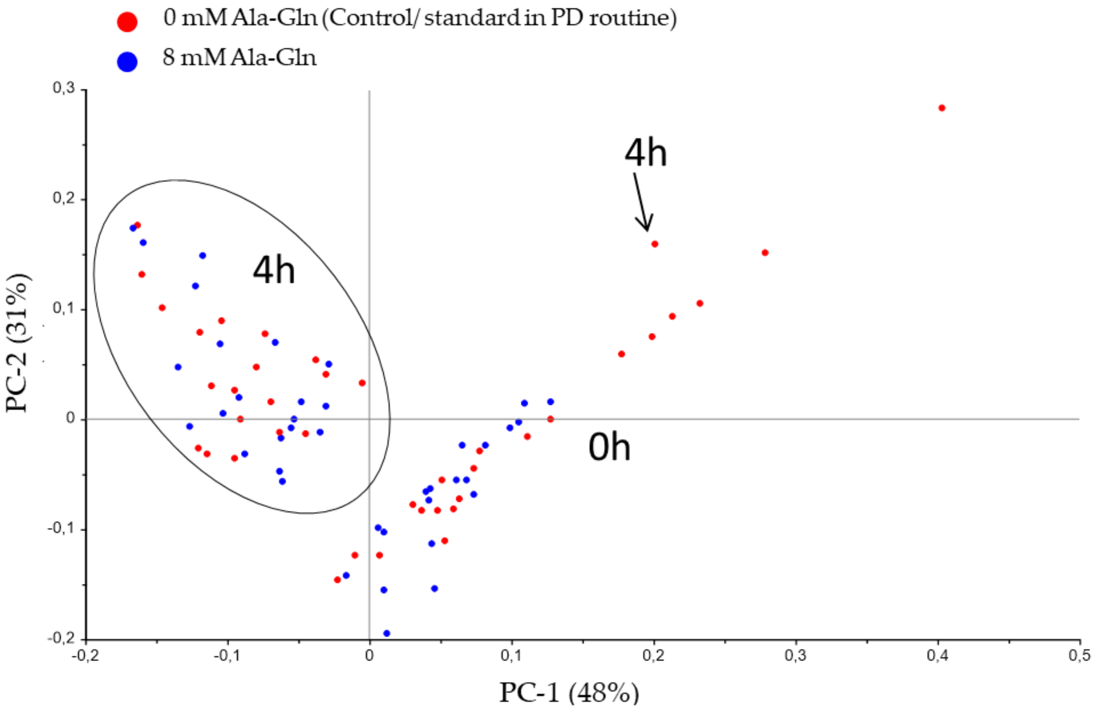

Unsupervised PCA score plot based on FTIR spectra recorded from patient PD fluid examining the polysaccharide spectral region (1200-800cm^{-1}) for data analysis . PC1 was plotted against PC2. Samples derived at time point 4h can be clearly separated from samples at time point 0h, but no discrimination was obtained between spectra derived from PD fluids of Ala-Gln treated and non-treated patients.

Figure S1. PCA score plot from FTIR data based on the polysaccharide spectral region (1200–800cm^{-1}). The equivalent score plot for the protein region is provided in Figure 3a.

Table S1. Confusion matrix incl. classification rates of patient-related data (balanced).

**Spectral range:**	**Protein region (1800-1500 cm^{-1})**				**Polysaccharide region (1200-800 cm^{-1})**			
**Classifier:**	**PCA-LDA**		**PCA-MDA**		**PCA-LDA**		**PCA-MDA**	
**Age (<60/ ≥60 years)**								
**control, 4h (n=20)**	Actual		Actual		Actual		Actual	
Predicted	≥60	<60	≥60	<60	≥60	<60	≥60	<60
≥60	8	2	10	0	10	1	10	0
<60	2	8	0	10	0	9	0	10
Accuracy %	80,0	80,0	100,0	100,0	100,0	90,0	100,0	100,0
**Overall accuracy %**		**80,0**		**100,0**		**95,0**		**100,0**
**all, 4h (n=40)**	Actual		Actual		Actual		Actual	
Predicted	≥60	<60	≥60	<60	≥60	<60	≥60	<60
≥60	13	5	13	6	12	3	14	10
<60	7	15	7	14	8	17	6	10
Accuracy %	65,0	75,0	65,0	70,0	60,0	85,0	70,0	50,0
**Overall accuracy %**		**70,0**		**67,5**		**72,5**		**60,0**
**Sex (female/ male)**								
**control, 4h (n=20)**	Actual		Actual		Actual		Actual	
Predicted	female	male	female	male	female	male	female	male
female	5	2	2	0	5	2	3	0
male	2	11	5	13	2	11	4	13
Accuracy %	71,43	84,62	28,57	100,00	71,43	84,62	42,86	100,00
**Overall accuracy %**		**78,0**		**64,3**		**78,0**		**71,4**
**all, 4h (n=40)**	Actual		Actual		Actual		Actual	
Predicted	female	male	female	male	female	male	female	male
female	8	14	8	4	8	14	5	8
male	8	10	8	20	8	10	11	16
Accuracy %	50,00	41,67	50,00	83,33	50,00	41,67	31,25	66,67
**Overall accuracy %**		**45,8**		**66,7**		**45,8**		**49,0**

Table S2. Confusion matrix incl. classification rates of PD-related data (balanced).

**Spectral range:**	**Protein region (1800-1500 cm^{-1})**				**Polysaccharide region (1200-800 cm^{-1})**			
**Classifier:**	**PCA-LDA**		**PCA-MDA**		**PCA-LDA**		**PCA-MDA**	
**Modality (CAPD/ APD-Cycler)**								
**control, 4h (n=20)**	Actual		Actual		Actual		Actual	
Predicted	CAPD	APD-Cycler	CAPD	APD-Cycler	CAPD	APD-Cycler	CAPD	APD-Cycler
CAPD	8	2	10	2	9	0	10	1
APD-Cycler	2	8	0	8	1	10	0	9
Accuracy %	80,0	80,0	100,0	80,0	90,0	100,0	100,0	90,0
**Overall accuracy %**		**80,0**		**90,0**		**95,0**		**95,0**
**all, 4h (n=40)**	Actual		Actual		Actual		Actual	
Predicted	CAPD	APD-Cycler	CAPD	APD-Cycler	CAPD	APD-Cycler	CAPD	APD-Cycler
CAPD	18	3	18	7	15	4	17	4
APD-Cycler	2	17	2	13	5	16	3	16
Accuracy %	90,0	85,0	90,0	65,0	75,0	80,0	85,0	80,0
**Overall accuracy %**		**87,5**		**77,5**		**77,5**		**82,5**
**Glucose/ Icodextrin**								
**control, 4h (n=20)**	Actual		Actual		Actual		Actual	
Predicted	Glucose	Icodextrin	Glucose	Icodextrin	Glucose	Icodextrin	Glucose	Icodextrin
Glucose	n.D.		n.D.		n.D.		n.D.	
Icodextrin								
Accuracy %								
**Overall accuracy %**								
**all, 4h (n=40)**	Actual		Actual		Actual		Actual	
Predicted	Glucose	Icodextrin	Glucose	Icodextrin	Glucose	Icodextrin	Glucose	Icodextrin
Glucose	25	4	32	8	28	2	32	8
Icodextrin	7	4	0	0	4	6	0	0
Accuracy %	78,13	50,00	100,00	0,00	87,50	75,00	100,00	0,00
**Overall accuracy %**		**64,1**		**50,0**		**81,3**		**50,0**
**Time on PD (<1/ ≥1 year)**								
**control, 4h (n=20)**	Actual		Actual		Actual		Actual	
Predicted	<1 year	≥1 year	<1 year	≥1 year	<1 year	≥1 year	<1 year	≥1 year
<1 year	n.D.		n.D.		n.D.		n.D.	
≥1 year								
Accuracy %								
**Overall accuracy %**								
**all, 4h (n=40)**	Actual		Actual		Actual		Actual	
Predicted	<1 year	≥1 year	<1 year	≥1 year	<1 year	≥1 year	<1 year	≥1 year
<1 year	6	7	0	9	5	5	0	2
≥1 year	4	23	10	21	5	25	10	28
Accuracy %	60,00	76,67	0,00	70,00	50,00	83,33	0,00	93,33
**Overall accuracy %**		**68,3**		**35,0**		**66,7**		**46,7**
**UrinVolOut (</≥1000 mL)**								
**control, 4h (n=20)**	Actual		Actual		Actual		Actual	
Predicted	<1000 mL	≥1000 mL	<1000 mL	≥1000 mL	<1000 mL	≥1000 mL	<1000 mL	≥1000 mL
<1000 mL	12	1	12	0	9	2	12	1
≥1000 mL	0	7	0	8	3	6	0	7
Accuracy %	100,00	87,50	100,00	100,00	75,00	75,00	100,00	87,50
**Overall accuracy %**		**93,8**		**100,0**		**75,0**		**93,8**
**all, 4h (n=40)**	Actual		Actual		Actual		Actual	
Predicted	<1000 mL	≥1000 mL	<1000 mL	≥1000 mL	<1000 mL	≥1000 mL	<1000 mL	≥1000 mL
<1000 mL	20	7	13	2	12	12	11	2
≥1000 mL	4	9	11	14	4	12	13	14
Accuracy %	83,33	56,25	54,17	87,50	75,00	50,00	45,83	87,50
**Overall accuracy %**		**69,8**		**70,8**		**62,5**		**66,7**

(continued - next page)

Table S2. *Cont.*

**Spectral range:**	**Protein region (1800-1500 cm^{-1})**				**Polysaccharide region (1200-800 cm^{-1})**			
**Classifier:**	**PCA-LDA**		**PCA-MDA**		**PCA-LDA**		**PCA-MDA**	
**UF (</≥550 mL)**								
**control, 4h (n=20)**	Actual		Actual		Actual		Actual	
Predicted	<550 mL	≥550 mL	<550 mL	≥550 mL	<550 mL	≥550 mL	<550 mL	≥550 mL
<550 mL	8	3	9	0	9	1	10	0
≥550 mL	2	7	1	10	1	9	0	10
Accuracy %	80,00	70,00	90,00	100,00	90,00	90,00	100,00	100,00
**Overall accuracy %**		**75,0**		**95,0**		**90,0**		**100,0**
**all, 4h (n=40)**	Actual		Actual		Actual		Actual	
Predicted	<550 mL	≥550 mL	<550 mL	≥550 mL	<550 mL	≥550 mL	<550 mL	≥550 mL
<550 mL	17	9	14	7	14	6	14	9
≥550 mL	5	9	8	11	8	12	8	9
Accuracy %	77,27	50,00	63,64	61,11	63,64	66,67	63,64	50,00
**Overall accuracy %**		**63,6**		**62,4**		**65,2**		**56,8**
**ResidualCl (</≥3 mL/min/1.73m^2)**								
**control, 4h (n=20)**	Actual		Actual		Actual		Actual	
Predicted	<3 *)	≥3 $^{\#}$)	<3 *)	≥3 $^{\#}$)	<3 *)	≥3 $^{\#}$)	<3 *)	≥3 $^{\#}$)
<3 mL/min/1.73m^2 *)	12	1	13	2	11	2	13	6
≥3 mL/min/1.73$m^{2\,\#}$)	1	6	0	5	2	5	0	1
Accuracy %	92,31	85,71	100,00	71,43	84,62	71,43	100,00	14,29
**Overall accuracy %**		**89,0**		**85,7**		**78,0**		**57,1**
**all, 4h (n=40)**	Actual		Actual		Actual		Actual	
Predicted	<3 *)	≥3 $^{\#}$)	<3 *)	≥3 $^{\#}$)	<3 *)	≥3 $^{\#}$)	<3 *)	≥3 $^{\#}$)
<3 mL/min/1.73m^2 *)	22	7	14	2	22	13	11	2
≥3 mL/min/1.73$m^{2\,\#}$)	3	8	11	13	3	2	14	13
Accuracy %	88,00	53,33	56,00	86,67	88,00	13,33	44,00	86,67
**Overall accuracy %**		**70,7**		**71,3**		**50,7**		**65,3**
**DP Crea (</≥ 0.8 (-))**								
**control, 4h (n=20)**	Actual		Actual		Actual		Actual	
Predicted	<0.8 (-)	≥0.8 (-)	<0.8 (-)	≥0.8 (-)	<0.8 (-)	≥0.8 (-)	<0.8 (-)	≥0.8 (-)
<0.8 (-)	9	1	10	0	11	1	11	0
≥0.8 (-)	2	8	1	9	0	8	0	9
Accuracy %	81,82	88,89	90,91	100,00	100,00	88,89	100,00	100,00
**Overall accuracy %**		**85,4**		**95,5**		**94,4**		**100,0**
**all, 4h (n=40)**	Actual		Actual		Actual		Actual	
Predicted	<0.8 (-)	≥0.8 (-)	<0.8 (-)	≥0.8 (-)	<0.8 (-)	≥0.8 (-)	<0.8 (-)	≥0.8 (-)
<0.8 (-)	20	4	21	8	19	5	19	3
≥0.8 (-)	5	11	4	7	6	10	6	12
Accuracy %	80,00	73,33	84,00	46,67	76,00	66,67	76,00	80,00
**Overall accuracy %**		**76,7**		**65,3**		**71,3**		**78,0**

Table S3. Confusion matrix incl. classification rates of immune system-related data (balanced).

**Spectral range:**	**Protein region (1800-1500 cm^{-1})**				**Polysaccharide region (1200-800 cm^{-1})**			
**Classifier:**	**PCA-LDA**		**PCA-MDA**		**PCA-LDA**		**PCA-MDA**	
**IL-8 (</≥3 pg/mL)**								
**control, 4h (n=20)**	Actual		Actual		Actual		Actual	
Predicted	≥3 pg/mL	<3 pg/mL	≥3 pg/mL	<3 pg/mL	≥3 pg/mL	<3 pg/mL	≥3 pg/mL	<3 pg/mL
≥3 pg/mL	6	2	11	3	6	2	11	3
<3 pg/mL	5	7	0	6	5	7	0	6
Accuracy %	54,5	77,8	100,0	66,7	54,5	77,8	100,0	66,7
**Overall accuracy %**		**66,2**		**83,3**		**66,2**		**83,3**
**all, 4h (n=40)**	Actual		Actual		Actual		Actual	
Predicted	≥3 pg/mL	<3 pg/mL	≥3 pg/mL	<3 pg/mL	≥3 pg/mL	<3 pg/mL	≥3 pg/mL	<3 pg/mL
≥3 pg/mL	12	7	23	11	15	6	24	13
<3 pg/mL	15	6	4	2	12	7	3	0
Accuracy %	44,4	46,2	85,2	15,4	55,6	53,8	88,9	0,0
**Overall accuracy %**		**45,3**		**50,3**		**54,7**		**44,4**
**IL-6 (</≥200 pg/mL)**								
**control, 4h (n=20)**	Actual		Actual		Actual		Actual	
Predicted	<200 pg/mL	≥200 pg/mL	<200 pg/mL	≥200 pg/mL	<200 pg/mL	≥200 pg/mL	<200 pg/mL	≥200 pg/mL
<200 pg/mL	13	2	13	7	11	2	13	7
≥200 pg/mL	0	5	0	0	2	5	0	0
Accuracy %	100,00	71,43	100,00	0,00	84,62	71,43	100,00	0,00
**Overall accuracy %**		**85,7**		**50,0**		**78,0**		**50,0**
**all, 4h (n=40)**	Actual		Actual		Actual		Actual	
Predicted	<200 pg/mL	≥200 pg/mL	<200 pg/mL	≥200 pg/mL	<200 pg/mL	≥200 pg/mL	<200 pg/mL	≥200 pg/mL
<200 pg/mL	21	4	23	8	24	6	23	8
≥200 pg/mL	7	8	5	4	4	6	5	4
Accuracy %	75,00	66,67	82,14	33,33	85,71	50,00	82,14	33,33
**Overall accuracy %**		**70,8**		**57,7**		**67,9**		**57,7**
**Hsp-72 (</≥0.5 AU)**								
**control, 4h (n=20)**	Actual		Actual		Actual		Actual	
Predicted	<0.5 AU	≥0.5 AU	<0.5 AU	≥0.5 AU	<0.5 AU	≥0.5 AU	<0.5 AU	≥0.5 AU
<0.5 AU	9	0	9	0	7	2	9	0
≥0.5 AU	0	11	0	11	2	9	0	11
Accuracy %	100,00	100,00	100,00	100,00	77,78	81,82	100,00	100,00
**Overall accuracy %**		**100,0**		**100,0**		**79,8**		**100,0**
**all, 4h (n=40)**	Actual		Actual		Actual		Actual	
Predicted	<0.5 AU	≥0.5 AU	<0.5 AU	≥0.5 AU	<0.5 AU	≥0.5 AU	<0.5 AU	≥0.5 AU
<0.5 AU	9	6	7	12	6	8	10	9
≥0.5 AU	8	17	10	11	11	15	7	14
Accuracy %	52,94	73,91	41,18	47,83	35,29	65,22	58,82	60,87
**Overall accuracy %**		**63,4**		**44,5**		**50,3**		**59,8**
**Previous peritonitis (yes/no)**								
**control, 4h (n=20)**	Actual		Actual		Actual		Actual	
Predicted	Yes	No	Yes	No	Yes	No	Yes	No
Yes	n.D.		n.D.		n.D.		n.D.	
No								
Accuracy %								
**Overall accuracy %**								
**all, 4h (n=40)**	Actual		Actual		Actual		Actual	
Predicted	Yes	No	Yes	No	Yes	No	Yes	No
Yes	4	10	0	1	6	8	1	2
No	6	20	10	29	4	22	9	28
Accuracy %	40,00	66,67	0,00	96,67	60,00	73,33	10,00	93,33
**Overall accuracy %**		**53,3**		**48,3**		**66,7**		**51,7**
